# Impact of metabolic comorbidity on the association between body mass index and health-related quality of life: a Scotland-wide cross-sectional study of 5,608 participants

**DOI:** 10.1186/1471-2458-12-143

**Published:** 2012-02-24

**Authors:** Zia Ul-Haq, Daniel F Mackay, Elisabeth Fenwick, Jill P Pell

**Affiliations:** 1 Institute for Health and Wellbeing, University of Glasgow, Glasgow G12 8RZ, UK; 2 Institute for Health and Wellbeing, University of Glasgow, Room 305, 1 Lilybank Gardens Glasgow G12 8RZ, UK

**Keywords:** Body mass index, Health-related quality of life, Obesity, Overweight, SF-12, Utility

## Abstract

**Background:**

The prevalence of obesity is rising in Scotland and globally. Overall, obesity is associated with increased morbidity, mortality and reduced health-related quality of life. Studies suggest that "healthy obesity" (obesity without metabolic comorbidity) may not be associated with morbidity or mortality. Its impact on health-related quality of life is unknown.

**Methods:**

We extracted data from the Scottish Health Survey on self-reported health-related quality of life, body mass index (BMI), demographic information and comorbidity. SF-12 responses were converted into an overall health utility score. Linear regression analyses were used to explore the association between BMI and health utility, stratified by the presence or absence of metabolic comorbidity (diabetes, hypertension, hypercholesterolemia or cardiovascular disease), and adjusted for potential confounders (age, sex and deprivation quintile).

**Results:**

Of the 5,608 individuals, 3,744 (66.8%) were either overweight or obese and 921 (16.4%) had metabolic comorbidity. There was an inverted U-shaped relationship whereby health utility was highest among overweight individuals and fell with increasing BMI. There was a significant interaction with metabolic comorbidity (*p *= 0.007). Individuals with metabolic comorbidty had lower utility scores and a steeper decline in utility with increasing BMI (morbidly obese, adjusted coefficient: -0.064, 95% CI -0.115, -0.012, *p *= 0.015 for metabolic comorbidity versus -0.042, 95% CI -0.067, -0.018, *p *= 0.001 for no metabolic comorbidity).

**Conclusions:**

The adverse impact of obesity on health-related quality of life is greater among individuals with metabolic comorbidity. However, increased BMI is associated with reduced health-related quality of life even in the absence of metabolic comorbidity, casting doubt on the notion of "healthy obesity".

## Background

According to the World Health Organisation (WHO), more than one in ten of the world's adult population are obese [1]. In Scotland, around two-thirds of adult men and more than one-half of adult women are either overweight or obese (http://www.scotland.gov.uk/Topics/Statistics/Browse/Health/TrendObesity) and, in common with other developed countries, the prevalence is increasing. Overall, obesity is associated with an increased risk of many conditions including hypertension, hypercholesterolemia, type II diabetes and cardiovascular disease [2-5]. It is also associated with reduced life-expectancy [6-9]. There is growing evidence that the association between obesity and fatal or non-fatal events is mediated via these other conditions and that isolated obesity may not be injurious to health. In the United States of America, around 29% of obese men and 45% of obese women (totalling 19.5 million individuals) do not have metabolic comorbid conditions [10]. They do not appear to be at increased risk of cardiovascular events [11], and it has been suggested that weight loss will not be beneficial and may even increase their risk of cardio-metabolic outcomes [10-15]. This had led to the term "healthy obesity." Overall, obesity is associated with anxiety, depression and impaired health-related quality of life [16-19]. Previous research suggests that deterioration in health-related quality of life in overweight and obese individuals may be due to the presence of comorbidity [20]. It is currently unknown whether isolated or "healthy" obesity is associated with the decline in health-related quality of life. In this study, we used data from a Scotland-wide survey to address this question by comparing the health-related quality of life across the BMI category of people in the presence and absence of metabolic comorbidity.

## Methods

### Data source

The Scottish Health Survey has been conducted at regular intervals, of 3-5 years, since 1995. The Survey uses multi-stage, stratified probability sampling to ensure a representative sample of the general population. The trained staff collected data via face to face interview (including age, sex, postcode of residence, lifestyle risk factors, medication, past medical history and current health) and measured weight, height and blood pressure and obtained blood samples for assays (including total cholesterol concentrations) (http://www.esds.ac.uk/government/shes/). We used an extract of data from the 2003 Survey, the focus of which was cardiovascular disease and risk factors.

### Inclusion criteria and definitions

Our analyses were restricted to participants aged ≥ 20 years and those included were categorised into three age groups: 20-44, 45-64 and ≥ 65 years. Postcode of residence was used to allocate individuals to a socioeconomic quintile of the general population using the 2004 Scottish Index of Multiple Deprivation (SIMD) (http://www.scotland.gov.uk/Publications/2005/01/20458/49127). The index is derived from 31 markers of deprivation relating to health, education, housing, current income, employment access and crime, that are applied to each postcode data zones. There are 6,505 data zones in Scotland with a mean population of 750. Body Mass Index (BMI) was categorised according to the World Health Organisation definition [21]: underweight (BMI < 18.5 kg/m^2^), normal weight (BMI 18.5-24.9 kg/m^2^), overweight (BMI 25.0-29.9 40 kg/m^2^), and obese (BMI 30.0-39.9 kg/m^2^), with the addition of a category for morbidly obese (BMI ≥ 40 kg/m^2^). Metabolic comorbidity was defined as the presence of one or more of the following conditions known to be associated with obesity: diabetes, hypertension, hypercholesterolemia or cardiovascular disease. Cardiovascular disease was defined as angina or a past history of stroke or myocardial infarction and was based on participants reporting diagnosis by a doctor. Hypertension was defined as a blood pressure measurement of ≥ 140/90 mmHg, or anti-hypertensive medication. Hypercholesterolaemia was defined as a total cholesterol concentration ≥ 5.2 mmol/L, or lipid-lowering medication. Smoking status was self-reported and classified as never, ex- or current smoker. Alcohol consumption was self-reported and categorised as never, ex-, sensible and excessive, with the cut-off between sensible and excessive drinking defined as more than 14 units/week for women and 21 units/week for men [20] The responses obtained from the SF-12 questionnaires were converted into a single utility score using an algorithm developed by Brazier and colleagues at the University of Sheffield (http://www.shef.ac.uk/scharr/sections/heds/mvh/sf-6d/revisions.html) [22].

### Statistical analyses

All statistical analyses were performed using Stata version 11.2 (Stata Corporation, College Station, Texas, USA). Categorical data were summarized using frequencies and percentages and groups were compared using chi-square tests, or chi-square tests for trend for ordinal data. We used univariate and multivariate linear regression models to examine the association between BMI category and utility score, adjusting for the potential confounding effects of age, sex, deprivation quintiles, smoking status and alcohol consumption. Normal weight was used as the referent category. We tested whether there was a statistically significant interaction with metabolic comorbidity and stratified the analyses by the presence of metabolic comorbidity. The robustness of standard errors was checked using the bootstrapping method.

## Results

Of the 10,470 individuals who participated in the Scottish Health Survey, 7,097 were aged ≥ 20 years. Of these 6,559 (92%) had sufficient data to calculate a utility score. Participants who completed the SF-12 instrument were not significantly different in terms of BMI category (*p *= 0.225) and sex (*p *= 0.197), but were younger (*p *< 0.001), less socioeconomically deprived, (*p *< 0.001), and more likely to have metabolic comorbidity (*p *< 0.001). Among the 6,559 participants with a utility score, 5,608 (86%) also had BMI recorded and they comprised the study population. These individuals were not significantly different in term of metabolic comorbidity (*p *= 0.582) but were younger (*p *< 0.001), more likely to be male (*p *< 0.001) and less socioeconomically deprived (*p *= 0.020).

Of the 5,608 individuals, 2,531 (45.1%) were men and the mean age was 50 years (standard deviation 16 years). Nine hundred and twenty one (16.4%) had metabolic comorbidity and the mean utility score was 0.80 (standard deviation 0.14). One thousand seven hundred and ninety seven (32.0%) were normal weight, 2,276 (40.6%) overweight, 1,319 (23.5%) obese, 149 (2.7%) morbidly obese, and 67 (1.2%) underweight. There were significant differences between the BMI categories in terms of age and sex (Table 1). The percentage belonging to the most deprived quintile increased significantly from normal weight to morbidly obese, as did the percentage with metabolic comorbidity (Table 1).

**Table 1:** Characteristics of participants by body mass index category

	Underweight	Normal weight	Overweight	Obese	Morbidly obese	^**‡**^***p*-value**	Overall
	N = 67	N = 1,797	N = 2,276	N = 1,319	N = 149		N = 5, 608
	N (%)	N (%)	N (%)	N (%)	N (%)		N (%)
**Age (years)**							
20-44	32 (47.8)	937 (52.1)	858 (37.7)	440 (33.4)	54 (36.2)	< 0.001	2,321 (41.4)
45-64	21 (31.3)	562 (31.3)	916 (40.3)	549 (41.6)	75 (50.3)		2,123 (37.9)
≥ 65	14 (20.9)	298 (16.6)	502 (22.1)	330 (25.0)	20 (13.4)		1, 164 (20.8)
**Sex**							
Male	26 (38.8)	688 (38.3)	1,183 (52.0)	598 (45.3)	36 (24.2)	< 0.001	2,531 (45.1)
Female	41 (61.2)	1,109 (61.7)	1,093 (48.0)	721 (54.7)	113 (75.8)		3,077 (54.9)
**Deprivation quintile**							
1 (Least deprived)	14 (20.9)	389 (21.7)	498 (21.9)	218 (16.5)	16 (10.7)	< 0.001	1,135 (20.2)
2	11 (16.4)	413 (23.0)	537 (23.6)	258 (19.6)	30 (20.1)		1,249 (22.3)
3	9 (13.4)	385 (21.4)	503 (22.1)	337 (25.6)	29 (19.5)		1,263 (22.5)
4	14 (20.9)	322 (17.9)	427 (18.8)	276 (20.9)	26 (17.6)		1,065 (19.0)
5 (Most deprived)	19 (28.4)	288 (16.0)	311 (13.7)	230 (17.4)	48 (32.2)		896 (16.0)
**Metabolic comorbidity**							
No	59 (88.1)	1,632 (90.8)	1,899 (83.4)	990 (75.0)	107 (71.8)	< 0.001	4,687 (83.6)
Yes	8 (12.0)	165 (9.2)	377 (16.6)	329 (25.0)	42 (28.2)		921 (16.4)
**Smoking status**							
Never smoker	21 (31.3)	748 (41.6)	1, 002 (44.0)	599 (45.4)	60 (40.3)	< 0.001	2, 430 (43.3)
Ex-smoker	7 (10.5)	409 (22.8)	737 (32.4)	447 (33.9)	56 (37.6)		1, 656 (29.5)
Current smoker	39 (58.2)	640 (35.6)	537 (23.6)	273 (20.7)	33 (22.2)		1, 522 (27.1)
**Drinking status**							
Never drinker	12 (17.9)	89 (5.0)	106 (4.7)	72 (5.5)	8 (5.4)	< 0.001	287 (5.1)
Ex-drinker	5 (7.5)	83 (4.6)	85 (3.7)	62 (4.7)	14 (9.4)		249 (4.4)
Sensible drinker*	39 (58.2)	1, 266 (70.5)	1, 560 (68.5)	936 (70.9)	100 (67.1)		3, 901 (69.5)
Excessive drinker	11 (16.4)	352 (19.7)	522 (23.0)	246 (18.7)	27 (18.1)		1, 158 (20.7)
Missing	0 (0)	7 (0.39)	3 (0.13)	3 (0.23)	0 (0)		13 (0.23)

^‡^chi-square tests for trend * < 21 units/week for men, < 14 units/week for women

**Figure 1:**
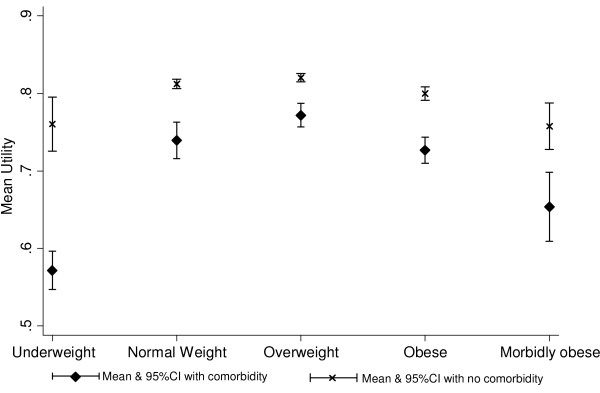
**Mean utility score by body mass index category and presence of metabolic comorbidity (unadjusted)**.

In relation to the association between BMI category and utility score, there was a significant interaction with metabolic comorbidity (*p *= 0.007). In every BMI category, the utility score was lower among those with metabolic comorbidity (Figure 1). Among both individuals with and without metabolic comorbidity, there was an inverted U-shaped relationship whereby health utility was highest among overweight individuals and fell with increasing BMI, with the decline steepest among those with metabolic comorbidity (Figure 1). Health related-quality of life was significantly reduced among obese individuals regardless of the presence or absence of metabolic comorbidity. After adjustment for the potential confounding effects of age, sex, deprivation smoking status and alcohol consumption, the utility score was non-significantly higher among overweight than normal weight individuals, irrespective of the presence of metabolic comorbidity (Table 2). Compared with normal weight individuals, utility scores were significantly lower among both morbidly obese and underweight individuals in both groups (Table 2).

**Table 2:** Linear regression analysis of the factors associated with utility score by presence or absence of metabolic comorbidity

		Univariate				Multivariate			
		No metabolic comorbidity		With metabolic comorbidity		No metabolic comorbidity		With metabolic comorbidity	
		Coefficient (95% CI)	P-value	Coefficient (95% CI)	P-value	Coefficient (95% CI)	P-value	Coefficient (95% CI)	P-value
**BMI category**	Underweight	-0.051 (-0.084, -0.018)	0.002	-0.167 (-0.275, -.059)	0.002	-0.036 (-0.069, -0.004)	0.027	-0.141 (-0.245, -0.037)	0.008
	Normal-weight*	-	-	-	-	-	-	-	-
	Overweight	0.008 (-0.000, 0.016)	0.059	0.032 (0.004, 0.060)	0.023	0.001 (-0.008, 0.009)	0.900	0.026 (-0.002, 0.053)	0.064
	Obese	-0.012 (-0.022, -0.002)	0.015	-0.012 (-0.041, 0.015)	0.380	-0.016 (-0.026, -0.006)	0.001	-0.015 (-0.043, 0.013)	0.290
	Morbidly obese	-0.054(-0.079, -0.029)	< 0.001	-0.085 (-0.137, -0.034)	0.001	-0.045 (-0.069, -0.020)	< 0.001	-0.077 (-0.128, -0.026)	0.003
**Age (yrs)**	20-44	0.002 (-0.005, 0.010)	0.500	0.001 (-0.036, 0.039)	0.924	0.005 (-0.002, 0.013)	0.190	0.007 (-0.030, 0.043)	0.714
	45-64	-	-	-	-	-	-	-	-
	≥ 65	-0.007 (-0.018, 0.003)	0.191	0.004 (-0.016, 0.025)	0.663	-0.009 (-0.020, 0.002)	0.106	0.004 (-0.017, 0.024)	0.718
**Sex**	Male*	-	-	-	-	-	-	-	-
	Female	-0.020 (-0.028, -0.013)	< 0.001	-0.009 (-0.029, 0.010)	0.357	-0.020 (-0.027, -0.013)	< 0. 001	-0.005 (-0.025, 0.015)	0.629
**Deprivation quintiles**									
	1(Least deprived)*	-	-	-	-	-	-	-	-
	2	-0.012 (-0.023, -0.001)	0.030	-0.016(-0.048, 0.015)	0.313	-0.008 (-0.019, 0.002)	0.132	-0.009 (-0.040, 0.021)	0.546
	3	-0.026 (-0.037, -0.015)	< 0.001	-0.053(-0.085, -0.021)	0.001	-0.019 (-0.030, -0.008)	0.001	-0.036 (-0.068, -0.004)	0.027
	4	-0.036 (-0.047, -0.024)	< 0.001	-0.070(-0.103, -0.038)	< 0.001	-0.026 (-0.038, -0.015)	< 0.001	-0.050 (-0.082, -0.017)	0.003
	5(Most deprived)	-0.070 (-0.082, -0.058)	< 0.001	-0.117(-0.150, -0.084)	< 0.001	-0.052 (-0.064, -0.040)	< 0.001	-0.084 (-0.117, -0.051)	< 0.001
**Smoking status**	Never smoker*	-	-	-	-	-	-	-	-
	Ex-smoker	-0.012 (-0.021, -0.004)	0.005	-0.031 (-0.053, -0.009)	0.006	-0.008 (-0.016, 0.001)	0.095	-0.031 (-0.053, -0.009)	0.006
	Current smoker	-0.051 (-0.060, -0.042)	< 0.001	-0.085 (-0.113, -0.058)	< 0.001	-0.041 (-0.050, -0.032)	< 0.001	-0.067 (-0.095, -0.038)	< 0.001
**Drinking status**	Never drinker*	-	-	-	-	-	-	-	-
	Ex-drinker	-0.058 (-0.083, -0.033)	< 0.001	-0.066 (-0.118, -0.013)	0.014	-0.050 (-0.075, -0.026)	< 0.001	-0.043 (-0.094, 0.008)	0.098
	Sensible drinker^‡^	0.010 (-0.008, 0.028)	0.281	0.033 (-0.002, 0.069)	0.067	0.003 (-0.014, 0.021)	0.710	0.032 (-0.02, 0.067)	0.068
	Excessive drinker	0.005 (-0.014, 0.024)	0.623	0.056 (0.015, 0.097)	0.007	-0.001 (-0.020, 0.018)	0.925	0.052 (0.010, 0.093)	0.014
	Missing	-0.059 (-0.136, 0.018)	0.133	0.029 (-0.186, 0.244)	0.793	-0.054 (-0.130, 0.021)	0.156	0.033 (-0.173, 0.239)	0.754

*Referent category, CI confidence interval, ^‡ ^< 21 units/week for men, < 14 units/week for women

## Discussion

Individuals with metabolic comorbidity have a poorer health-related quality of life than those without, irrespective of their BMI. However, health-related quality of life is significantly reduced among obese individuals even in the absence of metabolic comorbidity, suggesting that "healthy obesity" is a misnomer. Our findings are consistent with previous studies that have demonstrated reduced health-related quality of life among obese individuals [17,18,23-28]. However, these studies have only considered obese individuals as a whole. Historically, normal weight was associated with the lowest risk of cardiovascular diseases and type II diabetes, and the highest health-related quality of life [17,29]. This has changed over time, and our finding of non-significantly higher health-related quality of life among overweight individuals is consistent with other recent studies [28,30-32]. Previous studies have also shown poorer health among individuals with a low BMI [28,33,34]. This is likely to be due, in part, to reverse causation due to conditions other than those that we included in our definition of metabolic comorbidity.

There is a growing consensus that the increased risk of cardiometabolic events associated with obesity is mediated, largely, via the increased risk of intermediate conditions such as hypertension, hypercholesterolemia and type II diabetes [21]. A number of studies have identified a sub-group of obese individuals who do not develop these intermediate conditions [11]. They are not at significantly increased risk of cardiometabolic events, and weight loss does not improve their natural history [10-15]. These findings have led to the label "healthy" obesity.

Health extends beyond clinical events, to encompass psychological well-being. A number of studies have shown that health-related quality of life is reduced among obese individuals [35-38]. It was not previously known whether, as with clinical events, this risk was specific to obese individuals with metabolic comorbidity. Our study demonstrated that, whilst health-related quality of life was lower among individuals with metabolic comorbidity, it was nonetheless significantly reduced among obese individuals with no metabolic comorbidity.

The study used data from a large pan-Scotland survey representative of the general population. Due to incomplete data on BMI or utility score in 14% of participants, the study population was younger, more affluent and healthier than the overall survey population. However, this is unlikely to affect the generalisability of the results. Access to information on metabolic comorbidity enabled us to undertake sub-group analyses. BMI and blood pressure measurements were made by trained fieldworkers using standard operating procedures and the presence of hypercholesterolemia was based on blood assays. Presence of diabetes and cardiovascular disease were based on clinician diagnosis but reported by participants. Since the study was conducted retrospectively, this is unlikely to have led to reporting bias. In a cross-sectional study, a temporal relationship cannot be established. Therefore, reverse causation is possible. This is particularly so among individuals who are below normal weight in whom other conditions may be causing both poor health-related quality of life and weight loss. Survival bias may also occur in cross-sectional studies. Our findings should be corroborated within the context of a cohort study.

## Conclusions

Our study suggests that obesity is not only a risk for fatal and non-fatal clinical events but also reduced health-related quality of life, even in the absence of comorbid conditions. Our findings cast doubt on the notion of "healthy" obesity and reinforce the need for population and individual interventions to reverse the increasing prevalence of obesity.

## Abbreviations

BMI: body mass index; CI: confidence interval; N: number.

## Competing interests

The authors declare that they have no competing interests.

## Authors' contributions

JPP had the original concept. All of the authors agreed the methodology. ZUH and DFM performed the statistical analyses. All authors interpreted the results. ZUH drafted the manuscript. All authors fed back comments. All authors read and approved the final manuscript.

## Pre-publication history

The pre-publication history for this paper can be accessed here:

http://www.biomedcentral.com/1471-2458/12/143/prepub
